# Prognostic value of S1PR1 and its correlation with immune infiltrates in breast and lung cancers

**DOI:** 10.1186/s12885-020-07278-2

**Published:** 2020-08-15

**Authors:** Limei Zhong, Linling Xie, Zhiyong Yang, Lijuan Li, Shaohua Song, Donglin Cao, Yufeng Liu

**Affiliations:** 1 The First Affiliated Hospital, Guangzhou University of Chinese Medicine, No. 12 Airport Road, Baiyun District, Guangzhou, 510407 China; 2 Department of Laboratory Medicine, Guangdong Second Provincial General Hospital, No. 466 Xingang Middle Road, Haizhu District, Guangzhou, 510317 Guangdong Province China; 3 Lingnan Medical Research Center, Guangzhou University of Chinese Medicine, Guangzhou, 510407 China

**Keywords:** S1PR1, Breast cancer, Lung cancer, Tumor-infiltrating, Prognosis biomarker

## Abstract

**Background:**

Sphingosine-1-phosphate receptor (*S1PR1*) is involved in vascular development, a key process in tumorigenesis. This study aimed to evaluate its roles in tumor development and prognosis.

**Methods:**

S1PR1 expression levels were analyzed using TIMER and Oncomine database, and the prognostic significance of S1PR1 was assessed using PrognoScan and Kaplan-Meier plotter databases. The relationship between S1PR1 and tumor-infiltrated immune cells was analyzed using TIMER.

**Results:**

S1PR1 expression was remarkably lower in breast and lung cancer tissues than in the corresponding normal tissues. Lower expression was related to poor overall survival and disease-free survival in breast invasive carcinoma (BRCA), lung adenocarcinoma (LUAD), and lung squamous cell carcinoma (LUSC). A functional network analysis confirmed the function of S1PR1 in regulating vasculogenesis. In addition, S1PR1 levels were significantly negative with regard to the tumor purity of BRCA (*r* = − 0.508, *P =* 1.76e-66), LUAD (*r* = − 0.353, *P =* 6.05e-16), and LUSC (*r* = − 0.402, *P =* − 5.20e-20). Furthermore, S1PR1 levels were significantly positive with regard to infiltrating CD8^+^ (*r* = 0.38, *P =* 5.91e-35) and CD4^+^ T cells (*r* = 0.335, *P =* 1.03e-26), macrophages (*r* = 0.219, *P =* 3.67e-12), neutrophils (*r* = 0.168, *P =* 2.03e-7), and dendritic cells (DCs) (*r* = 0.208, *P =* 9.14e-11) in BRCA; S1PR1 levels were significantly positive with regard to CD8^+^ T cells (*r* = 0.308, *P =* 3.61e-12), macrophages (*r* = 0.376, *P =* 1.01e-17), neutrophils (*r* = 0.246, *P =* 4.15e-8), and DCs (*r* = 0.207, *P =* 4.16e-6) in LUAD; and positive with regard to B cells (*r* = 0.356, *P =* 1.57e-15), CD8^+^ (*r* = 0.459, *P =* 3.83e-26) and CD4^+^ T cells (*r* = 0.338, *P =* 3.98e-14), macrophages (*r* = 0.566, *P =* 2.61e-45), neutrophils (*r* = 0.453, *P =* 1.79e-25), and DCs (*r* = 0.56, *P =* 2.12e-40) in LUSC.

**Conclusions:**

S1PR1 levels are positively correlated with multiple immune markers in breast and lung cancer. These observed correlations between S1PR1 and the prognosis and immune cell infiltration provide a foundation for further research on its immunomodulatory role in cancer.

## Background

Sphingosine-1-phosphate (S1P), produced by sphingosine kinase (Sphk), is a biologically active signaling lipid [1]. S1P regulates vascular development and function, including vascular maturation [2, 3]. S1P receptor (S1PR1) is a biologically active sphingolipid metabolite that mediates S1P activity and promotes cell proliferation and survival [4, 5]. S1PR1 is widely expressed in vascular endothelial cells and is required for embryonic vascular development and maturation [6]. Estrogen (the growth-stimulating hormone in breast cancer cells) was shown to stimulate endothelial cell growth via S1PR1 [7, 8]. In the tumor microenvironment, S1P exhibits multiple functions: (a) it increases the survival of macrophages; (b) it serves as the “come-and-get-me” signal of dead cells, attracting and enhancing macrophage migration by combining with S1PR1; (c) it stimulates the polarization of TAM/M2 macrophages by activating S1PR1/2/4 [9–11]. Accumulating evidence demonstrated that tumor progression requires new blood vessel growth, which is achieved by producing angiogenic factors that can activate vascular endothelial cells [12]. Tumor cells release angiogenic stimuli, such as vascular endothelial growth factor (VEGF)-a, which leads to angiogenesis and tumor growth [13]. Studies have shown that S1PR1 inhibits VEGF signaling by promoting the interaction between VE-cadherin and VEGFR2, thereby inhibiting VEGF-induced vascular sprouting [14, 15].

However, the role of S1PR1 in tumorigenesis and its prognostic value are unclear. A preclinical study on human breast cancer cells found that S1PR1 antibody can enhance the cytotoxic and anti-proliferative effect of carboplatin on MDA-MB-231 and SK-BR-3 (HER2 subtype) cells, respectively [16]. Lei et al. found that S1PR1 signaling has tumor-suppressive effects and survival benefits in breast cancer [17]. Therefore, it is necessary to clarify the role of S1PR1 in tumor development and progression. Transcriptome analysis can be used to predict important issues, such as the intrinsic subtype of the primary tumor, tumor grade, drug reactivity, and recurrence risk [18–20].

Herein, we used Oncomine, Kaplan-Meier plotter, PrognoScan, UALCAN and GEPIA datasets to analyze S1PR1 expression and its relationship with the prognosis of cancer patients. Furthermore, we studied the correlation between S1PR1 and tumor-infiltrated immune cells in the tumor microenvironment using TIMER. Our results shed light on the important role of S1PR1 in breast and lung cancer, and determined that it is closely related to tumor immunity.

## Methods

### Oncomine database analysis

The Oncomine database (https://www.oncomine.org/resource/login.html) was used to evaluate the expression level of *S1PR1* in various types of cancers [21]. The thresholds were a *P*-value of 0.0001, fold change of 2.0 and data type was mRNA.

### PrognoScan database analysis

The PrognoScan database (www.prognoscan.org/) was used to test S1PR1 expression and survival in various types of cancers [22]. The threshold was an adjusted Cox *P*-value of < 0.05.

### C-BioPortal database analysis

c-BioPortal (http://cbioportal.org) contains multidimensional cancer genomics data sets [23]. *S1PR1* mutations and copy number variation (CNV) in breast and lung cancers were analyzed using c-BioPortal. The OncoPrint tab was used to obtain an overview of the genetic alterations for each sample.

### Kaplan-Meier plotter

Kaplan-Meier Plotter (https://kmplot.com/) was applied to assess the prognostic value of S1PR1. Grouped according to the median expression of S1PR1 (high vs low expression), all patients were analyzed for overall survival (OS) and progression-free survival (PFS), and Kaplan-Meier was used to draw a survival chart [24].

### Immune infiltrates analysis using the TIMER

TIMER 2.0 (https://cistrome.shinyapps.io/timer/) was used to analyze immune infiltrates across different types of cancer [25]. Especially, the expression of S1PR1 in different cancer types, and the correlation between the expression of S1PR1 and the abundance of immune invasion was determined. In addition, the correlation between S1PR1 expression and tumor infiltrating immune cell gene markers was also explored through related modules.

### Gene correlation analysis using GEPIA

GEPIA (http://gepia.cancer-pku.cn/index.html) was used to confirm the genes with significantly correlated expression levels in TIMER [26]. The Spearman method was used to determine the correlation coefficients. The tumor tissue datasets were used for analysis.

### LinkedOmics database analysis

The LinkedOmics database (http://www.linkedomics.org/login.php) was used to analyze S1PR1 co-expression based on Pearson’s correlation coefficients. The results were visually evaluated using volcano plots and heat maps. The function module of LinkedOmics was used to analyze gene ontology (GO) biological processes (BP) and Kyoto Encyclopedia of Genes and Genomes (KEGG) pathways by a gene set enrichment analysis (GSEA). The rank criterion was FDR < 0.05 and 500 simulations were performed [27].

### UALCAN database analysis

UALCAN (http://ualcan.path.uab.edu) included the Cancer Genome Atlas (TCGA) level RNA sequences. Clinical data from 31 cancer types were used to analyze the relative expression of genes in tumor and normal samples according to tumor stage, tumor grade or other clinicopathological characteristics [28].

### S1PR1 mRNA expression level analysis

Gene expression data of breast invasive carcinoma (BRCA), lung adenocarcinoma (LUAD), and lung squamous cell carcinoma (LUSC) in TCGA were downloaded in UCSC Xena (https://xenabrowser.net). S1PR1 mRNA expression level was compared between cancerous and normal tissue using Mann-Whitney test with *P* < 0.05 setting as a cut-off.

### Statistical analysis

Gene expression data in the Oncomine database was analyzed using *p*-value, fold change, and mRNA data type. The survival curves were generated via Kaplan-Meier plots and PrognoScan database are displayed with HR and P or Cox *P*-values from a log-rank test. Spearman correlation analysis was used to evaluate the correlation of gene expression in TIMER and LinkedOmics databases. *P* < 0.05 was considered statistically significant.

## Results

### S1PR1 mRNA expression levels in different types of human cancers

**Fig. 1:**
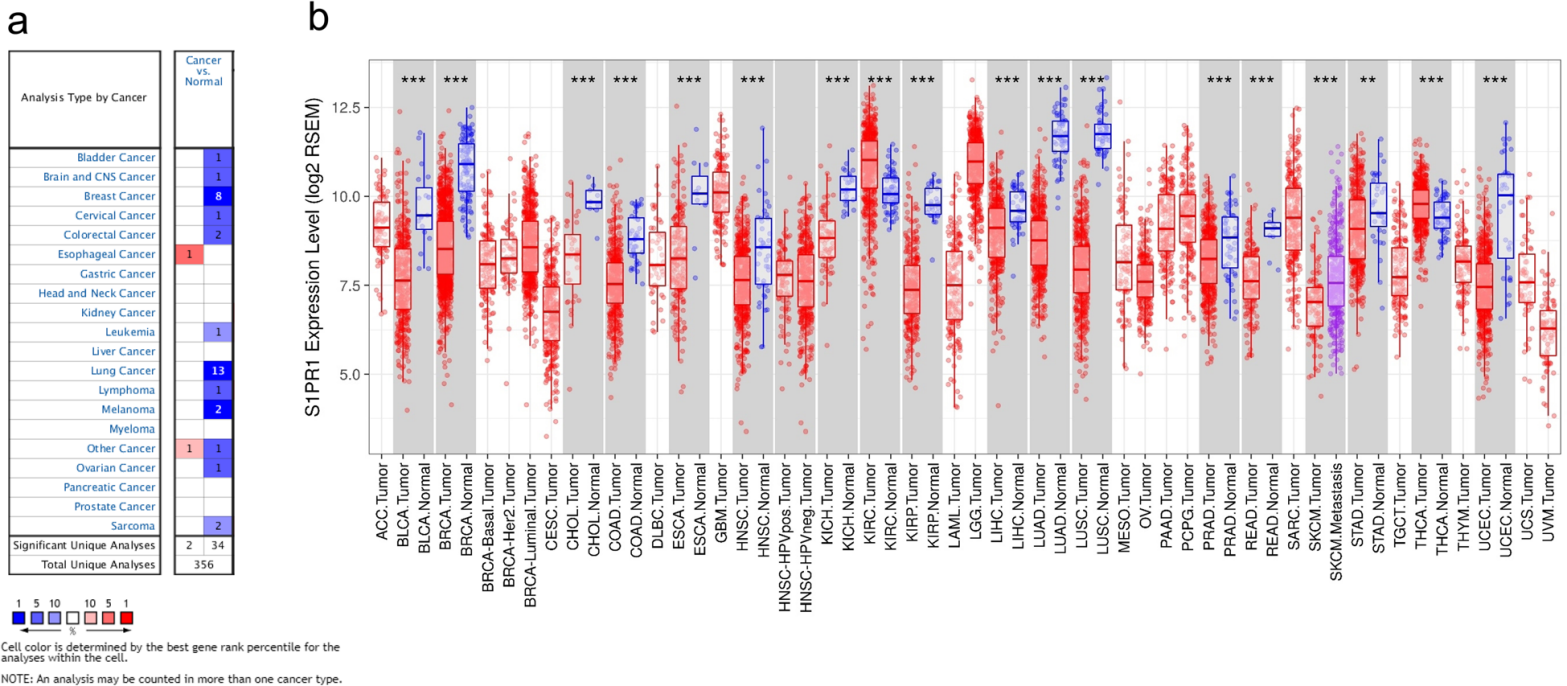
S1PR1 expression levels in different types of human cancers. **a** Differences in S1PR1 between cancer tissues and normal tissues based on data in the Oncomine database. (*P* = 1E-04, Fold change = 2, Data type = mRNA) (**b**) Human *S1PR1* expression levels in different tumor types from TCGA database were determined using TIMER 2.0. *P < 0.05, **P < 0.01, ***P < 0.001

The Oncomine database was used to analyze *S1PR1* mRNA levels in tumor tissues and normal tissues of various cancer types. S1PR1 expression was lower in most tumor tissues, including sarcoma, bladder, brain, central nervous system, breast, colorectal, leukemia, lung, myeloma, and ovarian cancer tissues, than in normal tissues (Fig. 1a). The mRNA-seq data from TCGA were analyzed using TIMER to verify these findings. Data from TCGA shown that the differential expression of S1PR1 between the tumor and adjacent normal tissues is shown in Fig. 1b. Compared with adjacent normal tissues, *S1PR1* expression was significantly reduced in bladder urothelial carcinoma (BLCA), BRCA, cholangiocarcinoma (CHOL), colon adenocarcinoma (COAD), esophageal carcinoma (ESCA), head and neck squamous cell carcinoma (HNSC), kidney chromophobe (KICH), kidney renal papillary cell carcinoma (KIRP), liver hepatocellular carcinoma (LIHC), LUAD, LUSC, prostate adenocarcinoma (PRAD), rectum adenocarcinoma (READ), skin cutaneous melanoma (SKCM), stomach adenocarcinoma (STAD), and uterine corpus endometrial carcinoma (UCEC). However, S1PR1 expression was significantly higher in kidney renal clear cell carcinoma (KIRC) and thyroid carcinoma (THCA) than in adjacent normal tissues (Fig. 1b). These data showed that alterations in S1PR1 expression depend on the tumor type, suggesting that this gene exerts diverse functions in various tumors.

### Prognostic evaluation of S1PR1 in cancers

**Fig. 2:**
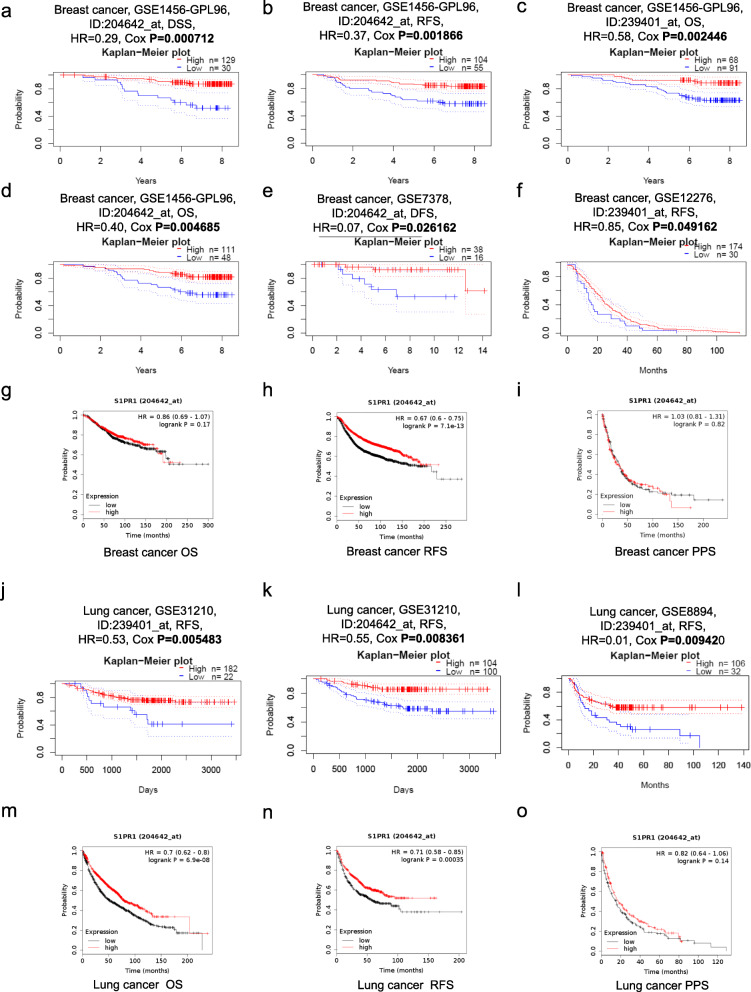
Prognostic value of S1PR1 in cancers. **a**–**f** Kaplan-Meier survival curves comparing high and low expression of S1PR1 in breast cancers using PrognoScan. Survival curves based on OS, DSS, and DFS in three breast cancer cohorts [GSE1456-GPL96 (*n* = 159), GSE7378 (*n* = 54) and GSE12276 (*n* = 204)]. **g**–**i** Survival curves for breast cancers based on mRNA-seq data from TCGA of Kaplan–Meier plotter databases. **j**–**l** Kaplan–Meier survival curves comparing high and low expression of S1PR1 in lung cancers using PrognoScan. Survival curves based on RFS in two three lung cancer cohorts [GSE31210 (*n* = 204) and GSE8894 (*n* = 138)]. **m**–**o** Survival curves for lung cancers based on mRNA-seq data from TCGA of Kaplan–Meier plotter databases. OS = Overall survival; RFS = Relapse-Free Survival; PPS = Post-progression survival; DSS = Disease-specific survival; DFS = Disease-free survival

We investigated whether S1PR1 expression is related to prognosis. The effect of S1PR1 expression on survival was evaluated by PrognoScan. Two probes (204642_at and 239401_at) matching S1PR1 were detected. Notably, in three breast cancer cohorts (GSE1456-GPL96, GSE7378, and GSE12276), low S1PR1 expression was significantly associated with a poorer prognosis breast cancer (Fig. 2a–f). We used the Kaplan-Meier plotter database to further examine the prognostic value of S1PR1 in breast cancer. Poor prognosis based on recurrence-free survival (RFS) in breast cancer was significantly correlated with low S1PR1 expression (HR = 0.67, *P* = 7.1e-13), but a significant correlation was not observed for overall survival (OS) (HR = 0.86, *P* = 0.17) and post-progression survival PPS (HR = 1.03, *P* = 0.82) (Fig. 2g–i). Its determined that the low expression of S1PR1 is an independent risk factor for poor prognosis of breast cancer. In addition, low S1PR1 expression was also related to poor prognosis in two cohorts of patients with lung cancer (GSE31210 and GSE8894), as determined using two probes (204642_at and 239401_at) (Fig. 2j–l). Kaplan-Meier plotter database also showed that low expression of S1PR1 was an independent risk factor for poor prognosis of lung cancer (overall survival, HR = 0.7, *P* = 6.9e-08; recurrence-free survival, HR = 0.71, *P* = 0.00035), but not related to post-progression survival in lung cancer (HR = 0.82, *P* = 0.14) (Fig. 2m–o).

Furthermore, we found that low S1PR1 expression was associated with a poor prognosis in patients with soft tissue, blood, and brain cancers (Fig. S1a–c). In contrast, low S1PR1 expression was an independent risk factor for a good prognosis in gastric cancer (Fig. S1d–g). These results confirmed the prognostic value of S1PR1 in specific types of cancer; both high and low S1PR1 expression was associated with prognosis depending on the type of cancer. Based on the consistent results for the associations between S1PR1 expression and survival in breast and lung cancers, we focused on the precise effects of S1PR1 in these two cancer types, as well as the underlying mechanisms.

### Correlations between clinical characteristics and S1PR1 expression in breast cancer and lung cancer

We used the Kaplan-Meier plotter to study the relationship between S1PR1 expression and clinical characteristics in patients with breast cancer and lung cancer. Low expression of S1PR1 was associated with worse overall survival (OS) in male and female patients with lung adenocarcinoma (*P* < 0.05) (Table 1). In particular, low S1PR1 mRNA expression was correlated with worse OS in stage 1 (*P* = 9.20E-13) and early-stage (AJCC stage M) (*P* = 0.013) lung cancer (Table 1). Low S1PR1 mRNA expression was related to poor OS in patients with (*P* = 0.023) or without (*P* = 0.00075) a smoking history (Table 1). In addition, low S1PR1 mRNA expression was related to worse OS in patients who did not receive chemotherapy or radiotherapy. These findings strongly suggest that low S1PR1 mRNA expression is correlated with poor OS in lung cancer (Table 1). In BRCA, low S1PR1 mRNA expression was related to poor OS in ER-positive or HER2-negative patients and in the luminal androgen receptor subtype (Table 2). Taken together, high expression of *S1PR1* could be considered a good prognostic indictor for breast and lung cancers depending on the clinical characteristics.

**Table 1:** Correlation between *S1PR1* mRNA expression and prognosis in lung cancer with respect to clinicopathological factors

Clinicopathological characteristics	Overall survival		
	N	Hazard ratio	*P*-value
Sex			
Female	715	0.72 (0.57–0.91)	**0.0064**
Male	1100	0.72 (0.61–0.84)	**4.90E-05**
Histology			
Adenocarcinoma	720	0.57 (0.45–0.73)	**5.90E-06**
Squamous cell carcinoma	524	0.85 (0.67–1.07)	0.1677
Stage			
1	577	0.35 (0.26–0.47)	**9.20E-13**
2	244	0.74 (0.51–1.07)	1.13E-01
3	70	1.03 (0.6–1.77)	9.20E-01
4	4	NA	NA
Grade			
I	201	1.19 (0.83–1.71)	0.34
II	310	0.83 (0.6–1.13)	0.23
III	77	0.61 (0.32–1.19)	0.15
AJCC stage T			
1	237	1.01 (0.76–1.34)	0.9527
2	389	0.77 (0.62–0.96)	0.019
3	81	1.47 (0.89–2.43)	0.13
4	46	0.98 (0.52–1.85	0.95
AJCC stage N			
0	781	0.85 (0.68–1.04)	0.12
1	56	1.78 (0.89–3.57)	0.098
2	111	1.27 (0.84–1.9)	0.2515
AJCC stage M			
0	681	0.77 (0.62–0.95)	**0.013**
1	10	NA	NA
Smoking history			
Exclude those never smoked	820	0.79 (0.64–0.94)	**0.023**
Only those never smoked	105	0.37 (0.21–0.68)	**0.00075**
Chemotherapy			
No	310	0.71 (0.51–1)	**0.046**
Yes	176	1.11 (0.74–1.67)	0.62
Radiotherapy			
No	271	0.69 (0.48–0.99)	**0.042**
Yes	70	1.04 (0.61–1.78)	0.8745

Bold values indicate *P* < 0.05; NA: none

**Table 2:** Correlations between *S1PR1* mRNA expression and clinical prognosis in breast cancer with respect to clinicopathological factors

Clinicopathological characteristics	Overall		
	N	Hazard ratio	*P*-value
ER status			
ER positive	2061	0.79 (0.67–0.94)	**0.0057**
ER negative	801	0.95 (0.7–1.18)	0.62
PR status			
PR positive	589	0.91 (0.64–1.29)	0.6024
PR negative	549	1.02 (0.76–1.36)	0.9124
HER2 status			
HER2 positive	252	1.13 (0.73–1.75)	0.5743
HER2 negative	800	0.75 (0.57–0.96)	**0.0247**
Intrinsic subtype			
Basal	241	1.23 (0.75–2.01)	0.41
Luminal A	611	0.75 (0.52–1.06)	0.1
Luminal B	433	0.97 (0.67–1.41)	0.88
HER2+	147	0.67 (0.35–1.28)	0.2235
Lymph node status			
Lymph node positive	313	0.94 (0.64–1.38)	0.75
Lymph node negative	594	1.07 (0.73–1.55)	0.74
Grade			
1	345	0.68 (0.4–1.15)	0.1461
2	901	0.94 (0.74–1.2)	0.63
3	903	0.93 (0.75–1.16)	0.5257
TP53 status			
Mutated	188	1.17 (0.73–1.88)	0.52
Wild type	273	0.81 (0.42–1.54)	0.52
Pietenpol subtype			
Basal-like 1	58	1.69 (0.55–5.17)	0.35
Basal-like 2	38	0.96 (0.28–3.34)	0.95
Immunomodulatory	100	1.67 (0.65–4.32)	0.28
Mesenchymal	73	0.79 (0.36–1.73)	0.56
Mesenchymal stem -like	19	NA	NA
Luminal androgen receptor	203	0.46 (0.3–0.71)	**0.0002**
Systemically untreated patients			
Include	1402	0.86 (0.69–1.07)	0.17
Exclude	3951	0.67 (0.6–0.75)	**7.1E-13**

Bold values indicate *P* < 0.05; NA: none

### Decreased expression of S1PR1 in breast cancer and lung cancer patients

**Fig. 3:**
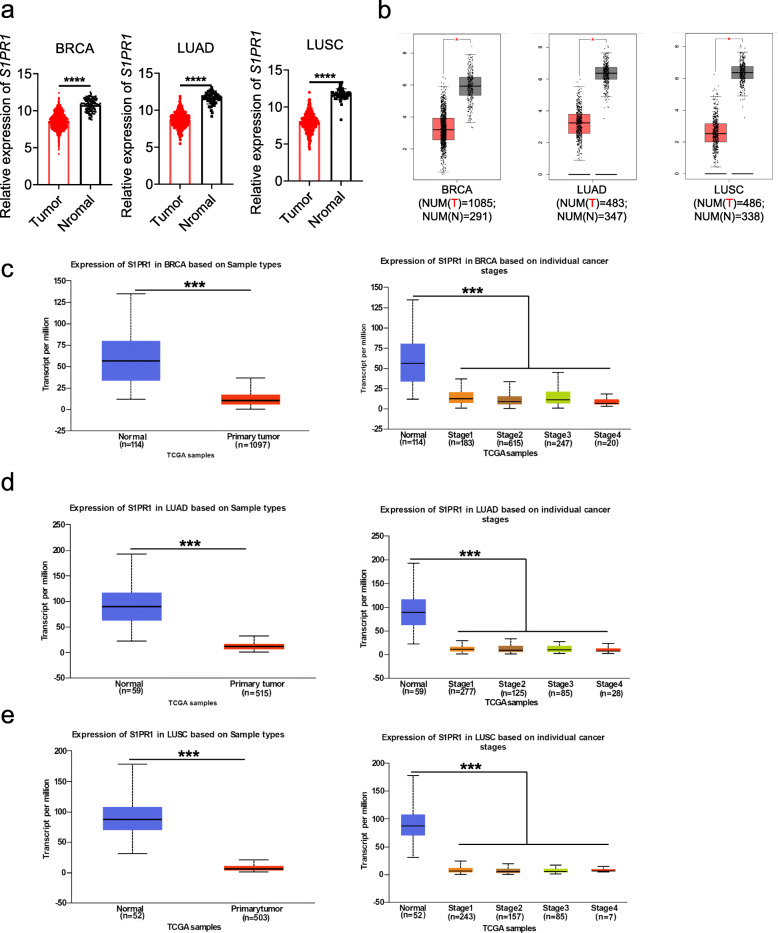
Decreased expression of S1PR1 in breast and lung cancer patients (**a**) Gene expression data of breast invasive carcinoma (BRCA), lung adenocarcinoma (LUAD), and lung squamous cell carcinoma (LUSC) in TCGA were downloaded in UCSC Xena. S1PR1 mRNA expression level was compared between cancerous and normal tissue using Mann-Whitney test with *P* < 0.05 setting as cut-off. **b** The expression of S1PR1 in BRCA, LUAD, and LUSC were analysis using GEPIA. T: tumor, N: normal tissue, NUM = number. **c**–**e** S1PR1 mRNA expression level was expressed as box plots using the UALCAN database. mRNA expression of S1PR1 in normal control and BRCA, LUAD, and LUSC tumors: (Left) primary tumors, (Right) individual cancer stage. *P < 0.05, **P < 0.01, ***P < 0.001

We further analyzed the expression of S1PR1 in breast and lung cancers. Gene expression data of breast invasive carcinoma (BRCA), lung adenocarcinoma (LUAD) and lung squamous cell carcinoma (LUSC) in TCGA were downloaded and *S1PR1* mRNA expression level was compared between tumor and normal tissue. As shown in Fig. 3a, the expression of S1PR1 was significantly decreased in tumor tissues of BRCA, LUAD and LUSC (Fig. 3a). In comparison with normal control tissues, breast cancer and lung cancer tissues presented lower expression of S1PR1, which was also observed by GEPIA analysis (Fig. 3b). Furthermore, we analyzed TCGA data using the UALCAN database. Compared to normal tissues, S1PR1 mRNA expression was significantly decreased in primary tumors and tumor stages (stage 1, stage 2, stage 3, and stage 4) of BRCA, LUAD, and LUSC (Fig. 3c–e). Taken together, these data confirmed the down-regulation of S1PR1 expression in breast cancer and lung cancer patients.

### Regulators of S1PR1 in breast cancer and lung cancer

**Fig. 4:**
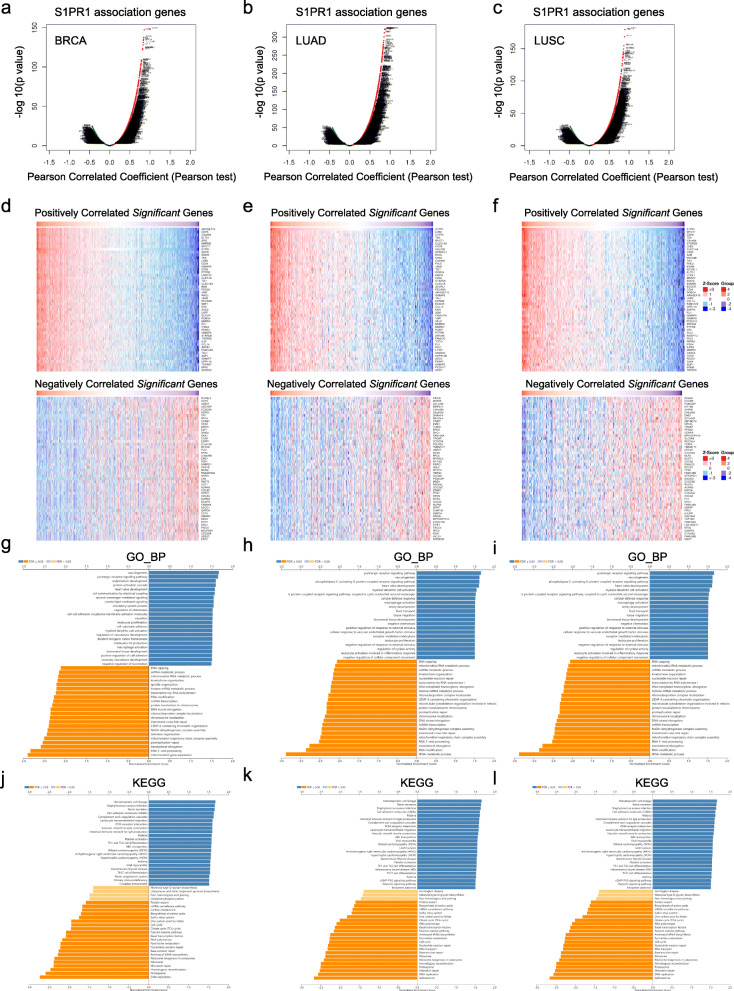
*S1PR1* co-expression genes in breast and lung cancer. **a**–**c** The *S1PR1* highly correlated genes identified by Pearson test in BRCA **(a)**, LUAD **(b)**, and LUSC **(c)**. **d**–**f** The heat map shows that in BRCA (**d**), LUAD (**e**), and LUSC (**f**), the first 50 genes are positively (red) and negatively (blue) correlated with *S1PR1*. **g**–**i** Significantly enriched GO annotations of *S1PR1* in BRCA (g), LUAD (h), and LUSC (i). **j**–**l** Significantly enriched KEGG pathways of *S1PR1* in BRCA (j), LUAD (**k**), and LUSC (**l**)

We used the LinkedOmics function module to detect the S1PR1 regulatory network to further understand the biological role of S1PR1 in breast cancer and lung cancer. Figure 4a–c shows genes with significantly positive (dark red dots) and negative (dark green dots) correlations with S1PR1 (false discovery rate, FDR < 0.01). The top 50 positively and negatively related genes are shown in a heat map in Fig. 4d–f. A Gene Ontology (GO)-based gene set enrichment analysis (GSEA) showed that genes that are co-expressed with S1PR1 are enriched for vasculogenesis and the purinergic receptor signaling pathway, while genes related to mitochondria and RNA transcript processing were inhibited in breast cancer (Fig. 4g). Similarly, GO annotation results showed that genes co-expressed with S1PR1 are primarily associated with vasculogenesis, the purinergic receptor signaling pathway, and the phospholipase C-activating G protein coupled receptor signaling pathway, while tRNA metabolic process, RNA modification, and RNA transcript processing were inhibited in lung cancer (Fig. 4h–i). A KEGG pathway analysis showed enrichment for hematopoietic cell lineage, *Staphylococcus aureus* infection, and renin secretion pathways in both breast cancer and lung cancer. Spliceosome, DNA replication, and proteasome pathways were inhibited in both tumor types (Fig. 4j-l). These results suggest that S1PR1 contributes to various processes in tumor development at least partially through regulate vasculogenesis.

### Genomic alterations in S1PR1 in breast cancer and lung cancer

**Fig. 5:**
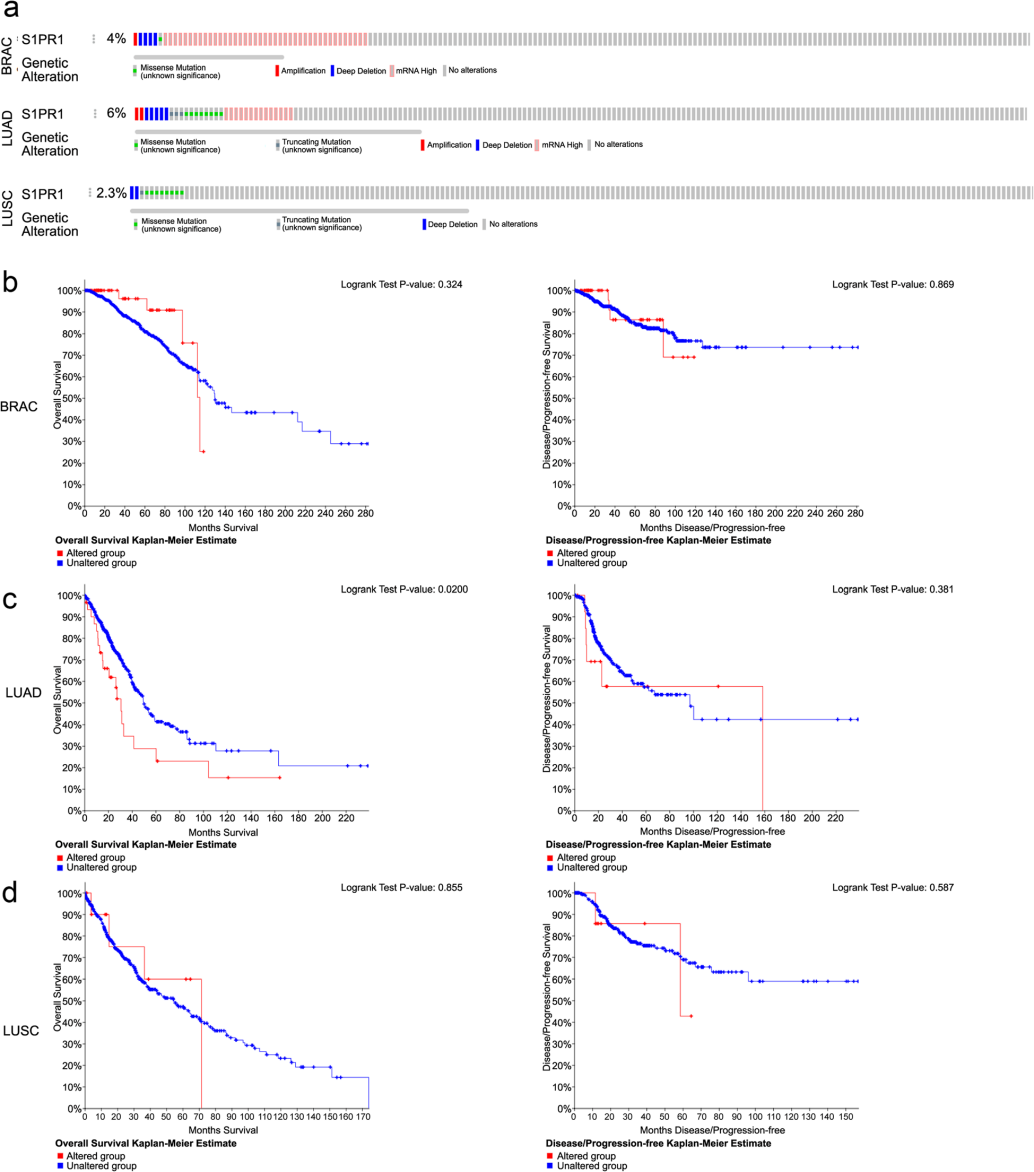
*S1PR1* genomic alterations in breast and lung cancer. **a** OncoPrint of *S1PR1* alterations in BRCA, LUAD, and LUSC. Different types of genetic alterations highlighted in different colors. **b**–**d** The relationship between genetic alterations and *S1PR1* (OS/DFS) in BRCA (b), LUAD (c), and LUSC (d). Logrank test was used in analysis of OS/DFS

cBioPortal database was used to determine the types and frequencies of S1PR1 alterations in BRCA, LUAD, and LUSC. *S1PR1* was altered in 4% of patients with BRCA. These alterations included mRNA missense mutations, amplifications, and deletions (Fig. 5a). *S1PR1* was altered in 6% of patients with LUAD and 2.3% of patients with LUSC, including mRNA missense mutations, truncating mutations, amplifications, and deletions (Fig. 5a). Moreover, S1PR1 CNV was associated with OS in LUAD but not with OS or DFS in BRCA and LUSC (Fig. 5b–d). These results suggest that mutations in S1PR1 are associated with prognosis in LUAD.

### Relationship between immune and S1PR1 expression in breast cancer and lung cancer

**Fig. 6:**
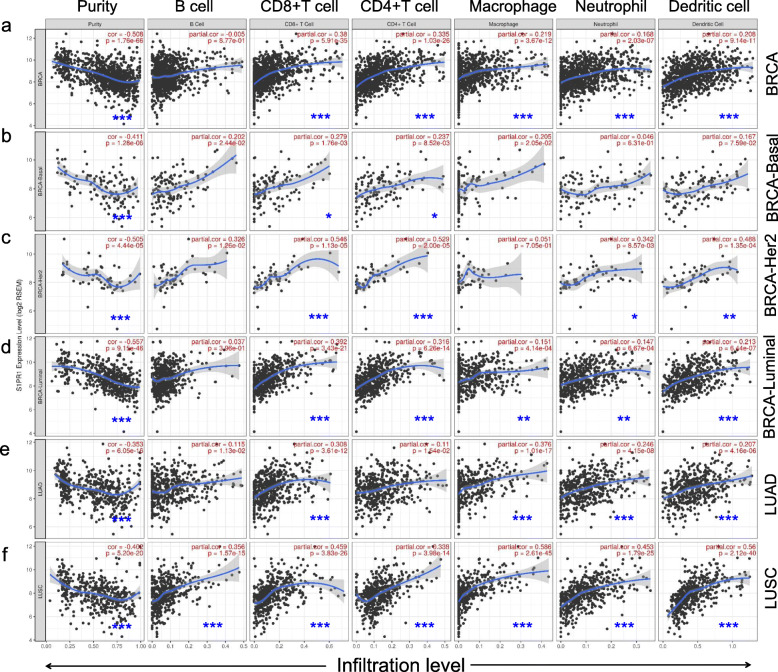
Correlations between S1PR1 expression and immune infiltration levels in breast and lung cancer. **a** S1PR1 expression was significantly negatively related to tumor purity and significantly positively correlated with infiltrating levels of CD8+ T cells, CD4+ T cells, macrophages, neutrophils, and dendritic cells in BRCA (*n* = 1093). **b** S1PR1 expression was significantly negatively related to tumor purity and was significant positively correlated with infiltrating levels of CD8+ T cells, CD4+ T cells, and dendritic cells in BRCA-Basal (*n* = 139). **c** S1PR1 expression was significantly negatively related to tumor purity and was significantly positively correlated with infiltrating levels of CD8+ T cells, CD4+ T cells, neutrophils, and dendritic cells in BRCA-Her2 (*n* = 67). **d** S1PR1 expression was significantly negatively related to tumor purity and was significantly positively correlated with infiltrating levels of CD8+ T cells, CD4+ T cells, macrophages, neutrophils, and dendritic cells in BRCA-Luminal (*n* = 611). **e** S1PR1 expression was significantly negatively related to tumor purity and was significantly positively correlated with infiltrating levels of CD8+ T cells, macrophages, neutrophils, and dendritic cells in LUAD (*n* = 457). **f** S1PR1 expression was significantly negatively related to tumor purity and was significant positively correlated with infiltrating levels of B cells, CD8+ T cells, CD4+ T cells, macrophages, neutrophils, and dendritic cells in LUSC (*n* = 457). Spearman’s correlation coefficients were used for analyses. * *P* < 0.01; ** *P* < 0.001; *** *P* < 0.0001

Tumor infiltrating lymphocytes (TIL) are lymphocytes that leave the blood circulation and migrate to the vicinity of the tumor. The amount of TIL in the tumor is an important indicator to predict the prognosis of cancer patients and the response to immunotherapy [29, 30]. Tumor purity is a key factor in analyses of immune infiltration by genomic approaches [31]. Therefore, we use TIMER to investigate whether the expression of S1PR1 in breast cancer and lung cancer is related to immune infiltration. We found a significant negative correlation between the S1PR1 expression level and tumor purity in both breast cancer and lung cancer (Fig. 6a–f, Left). S1PR1 was a determinant of immune infiltration in BRCA (tumor purity; *r* = − 0.508, *P* = 1.76e-66), including subtypes of BRCA (BRCA-Basal: *r* = − 0.5411, *P* = 1.28e-06; BRCA-Her2: *r* = − 0.505, *P* = 4.44e-06 and BRCA-Luminal: *r* = − 0.557, *P* = 9.15e-46). S1PR1 was related to immune infiltration in lung cancer, including LUAD (tumor purity; *r* = − 0.353, *P* = 6.05e-16) and LUSC (tumor purity; *r* = − 0.402, *P* = 5.20e-20).

Furthermore, the relationship between S1PR1 and specific immune infiltrates in breast cancer and lung cancer were analyzed. The S1PR1 expression level was significantly positively correlated with levels of infiltrating CD8^+^ T cells (*r* = 0.38, *P* = 5.97e-35), CD4^+^ T cells (*r* = 0.335, *P* = 1.03e-26), macrophages (*r* = 0.219, *P* = 3.67e-12), neutrophils (*r* = 0.168 *P* = 2.03e-07), and DCs (*r* = 0.208, *P* = 9.14e-11) in BRCA (Fig. 6a). In BRCA-Basal, there were slight positive correlations between S1PR1 expression levels and levels of infiltrating CD8^+^ T cells (*r* = 0.279, *P* = 1.76e-03) and CD4^+^ T cells (*r* = 0.237, *P* = 8.52e-03). Similarly, there were positive correlations with infiltrating levels of CD8^+^ T cells (*r* = 0.546, *P* = 1.13e-05), CD4^+^ T cells (*r* = 0.529, *P* = 2.00e-05), neutrophils (*r* = 0.342, *P* = 8.57e-03), and DCs (*r* = 0.488, *P* = 1.35e-04) in BRCA-Her2. S1PR1 expression levels were positively correlated with levels of infiltrating CD8^+^ T cells (*r* = 0.147, *P* = 3.43e-21), CD4^+^ T cells (*r* = 0.316, *P* = 6.26e-14), macrophages (*r* = 0.151, *P* = 4.14e-04), neutrophils (*r* = 0.147, *P* = 6.67e-04), and DCs (*r* = 0.213, *P* = 6.44e-07) in BRCA-Luminal tumors (Fig. 6a). We also found that S1PR1 expression levels were positively correlated with levels of infiltrating CD8^+^ T cells (*r* = 0.308, *P* = 3.61e-12), macrophages (*r* = 0.376, *P* = 1.01e-17), neutrophils (*r* = 0.246, *P* = 4.15e-08), and DCs (*r* = 0.207, *P* = 4.16e-06) in LUAD. In addition, there were positive correlations with levels of infiltrating B cells (*r* = 0.358, *P* = 1.27e-15), CD8+ T cells (*r* = 0.459, *P* = 3.83e-26), CD4^+^ T cells (*r* = 0.338, *P* = 3.98e-14), macrophages (*r* = 0.586, *P* = 2.61e-45), neutrophils (*r* = 0.453, *P* = 1.79e-25), and DCs (*r* = 0.56, *P* = 2.12e-40) in LUSC. These results strongly suggest that S1PR1 plays a special role in the immune infiltration of breast and lung cancers, and has a particularly strong effect on T cells, macrophages, neutrophils and DCs. These observed correlations between S1PR1 and various types of immune cells in breast and lung cancers indicated that S1PR1 may have high prognostic value.

### Correlations between S1PR1 expression and immune markers

**Fig. 7:**
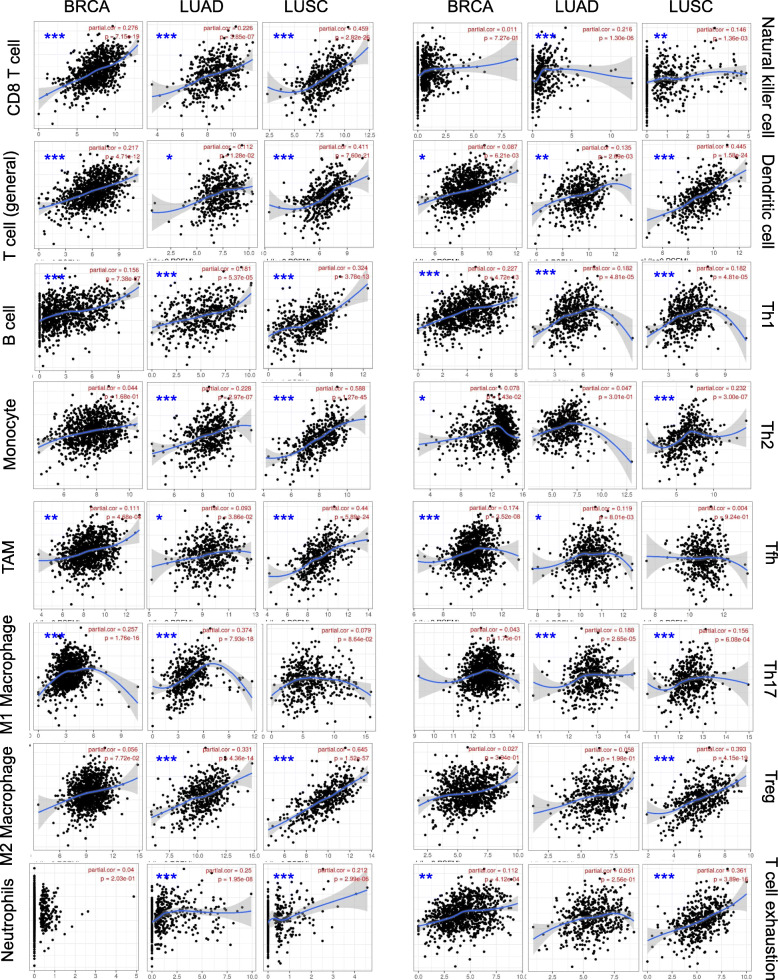
Correlations between S1PR1 expression and immune markers. Correlations between S1PR1 expression with markers of immune cells CD8+ T cell, T cell (general), B cells, monocytes, TAM, M1 macrophages, M2 macrophages, neutrophils, natural killer cells, dendritic cells, Th1, Th2, Tfh, Th17, Treg, and T cell exhaustion in BRCA, LUAD, and LUSC using TIMER 2.0

We further evaluated the correlations between S1PR1 and markers of various immune cells in breast cancer and lung cancer using TIMER (Table 3) and GEPIA databases (Table S1). The correlations between S1PR1 expression and immune marker genes for different immune cell populations, including CD8^+^ T cells, T cells (general), B cells, monocytes, TAMs, M1, and M2 macrophages, neutrophils, NK cells, DCs, and various functional T cells, such as Th1 cells, Th2 cells, Tfh cells, Th17 cells, and Tregs, as well as exhausted T cells were analyzed by TIMER. After adjusting for tumor purity, S1PR1 expression levels were significantly positively correlated with marker sets for various immune cells, except for NK cells, Th17, and T cell exhaustion in BRCA (Table 3 and Fig. 7). However, S1PR1 expression levels were highly positively correlated with most immune marker sets and both T cell populations and exhausted T cells in LUAD and LUSC (Table 3 and Fig. 7). We further analyzed the correlation between S1PR1 expression and the markers using the GEPIA database, including data for BRCA, LUAD, and LUSC. The results for correlations between S1PR1 and markers of immune infiltrating cells were similar to those of the TIMER analysis (Table S1). This further confirms that S1PR1 is significantly related to immune infiltrating cells in lung and breast cancer, suggesting that high levels of S1PR1 could induce immune activity in the lung and breast cancer microenvironment.

**Table 3:** Correlations between S1PR1 and related genes and markers of immune cells, as evaluated using TIMER

		BRCA			LUAD			LUSC		
Description	Gene markers	Purity			Purity			Purity		
	varX	cor	p		cor	p		cor	p	
CD8+ T cell	CD8A	0.267	1.26E-17	***	0.166	2.19E-04	**	0.411	6.51E-21	***
	CD8B	0.176	2.42E-08	***	0.108	1.66E-02		0.378	1.22E-17	***
T cell (general)	CD3D	0.217	4.71E-12	***	0.112	1.28E-02		0.411	7.60E-21	***
	CD3E	0.276	7.15E-19	***	0.226	8.85E-07	***	0.459	2.82E-26	***
	CD2	0.202	3.20E-10	***	0.159	4.00E-04	**	0.438	7.99E-24	***
B cell	CD19	0.156	7.38E-07	***	0.181	5.37E-05	***	0.324	3.78E-13	***
	CD79A	0.177	1.98E-08	***	0.172	1.21E-04	**	0.325	3.29E-13	***
Monocyte	CD86	0.044	1.28E-01		0.228	2.97E-07	***	0.588	1.27E-45	***
	CD115 (CSF1R)	0.202	1.29E-10	***	0.264	3.10E-08	***	0.64	2.67E-56	***
TAM	CCL2	0.111	4.68E-04	**	0.093	3.86E-02		0.44	5.89E-24	***
	CD68	0.023	4.63E-01		0.289	5.86E-11	***	0.494	1.18E-30	***
	IL10	0.055	8.35E-02		0.27	1.10E-09	***	0.534	1.49E-36	***
M1 Macrophage	INOS (NOS2)	0.257	1.76E-16	***	0.374	7.93E-18	***	0.079	8.64E-02	
	IRF5	0.016	6.18E-01		−0.042	3.55E-01		−0.036	4.31E-01	
	COX2 (PTGS2)	0.338	4.90E-28	***	0.095	3.58E-02		0.214	2.37E-06	***
M2 Macrophage	CD163	0.056	7.72E-02		0.331	4.36E-14	***	0.645	1.52E-57	***
	VSIG4	0.08	1.14E-02		0.271	9.75E-10	***	0.625	4.77E-53	***
	MS4A4A	0.23	1.96E-13	***	0.365	5.39E-17	***	0.628	9.28E-54	***
Neutrophils	CD66b (CEACAM8)	0.04	2.03E-01		0.25	1.95E-08	***	0.212	2.99E-06	***
	CD11b (ITGAM)	0.007	8.24E-01		0.199	8.16E-06	***	0.491	2.66E-30	***
	CCR7	0.316	1.55E-24	***	0.321	2.57E-13	***	0.514	1.70E-33	***
Natural killer cell	KIR2DL1	0.011	7.27E-01		0.216	1.30E-06	***	0.146	1.36E-03	*
	KIR2DL3	0.051	1.10E-01		0.148	9.96E-04	**	0.233	2.63E-07	***
	KIR2DL4	−0.027	3.95E-01		−0.03	5.06E-01		0.152	8.45E-04	**
	KIR3DL1	0.095	2.63E-03	*	0.174	1.04E-04	**	0.295	4.85E-11	***
	KIR3DL2	0.068	3.19E-02		0.077	8.79E-02		0.217	1.68E-06	***
	KIR3DL3	−0.005	8.75E-01		0.025	5.81E-01		0.044	3.43E-01	
	KIR2DS4	0.035	2.68E-01		0.119	8.34E-03	*	0.221	1.05E-06	***
Dendritic cell	HLA-DPB1	0.237	3.89E-14	***	0.261	4.13E-09	***	0.621	3.86E-52	**
	HLA-DQB1	0.073	2.11E-02		0.089	4.79E-02		0.4	8.84E-20	***
	HLA-DRA	0.156	7.17E-07	***	0.219	8.69E-07	***	0.603	1.29E-48	**
	HLA-DPA1	0.21	2.26E-11	***	0.225	4.53E-07	***	0.622	1.87E-52	**
	BDCA-1(CD1C)	0.461	1.76E-53	***	0.271	1.00E-09	***	0.438	8.69E-24	***
	BDCA-4(NRP1)	0.484	1.58E-59	***	0.174	1.07E-04	**	0.473	6.69E-28	***
	CD11c (ITGAX)	0.087	6.21E-03	*	0.135	2.69E-03	***	0.445	1.58E-24	***
Th1	T-bet (TBX21)	0.227	4.72E-13	***	0.182	4.81E-05	***	0.403	5.17E-20	***
	STAT4	0.277	5.92E-19	***	0.131	3.66E-03	***	0.504	4.73E-32	***
	STAT1	0.116	2.61E-04	**	−0.046	3.10E-01		0.177	1.03E-04	**
	IFN-g (IFNG)	0.009	7.84E-01	***	−0.076	9.13E-02		0.108	1.85E-02	
	TNF-a (TNF)	0.193	8.08E+ 10	***	−0.076	9.30E-02		0.069	1.34E-01	
Th2	GATA3	0.078	1.43E-02		0.047	3.01E-01		0.232	3.00E-07	***
	STAT6	0.225	6.69E-13	***	0.138	2.20E-03	*	0.022	6.25E-01	
	STAT5A	0.165	1.81E-07	***	0.248	2.27E-08	***	0.413	4.22E-21	***
	IL13	0.048	1.27E-01		0.071	1.15E-01		0.199	1.20E-05	***
Tfh	BCL6	0.174	3.52E-08	***	0.119	8.01E-03	*	0.004	9.24E-01	
	IL21	0.001	9.77E-01		0.054	2.34E-01		0.207	4.92E-06	***
Th17	STAT3	0.043	1.75E-01		0.188	2.65E-05	***	0.158	6.09E-04	**
	IL17A	−0.053	9.29E-02		0.033	4.62E-01		−0.038	4.09E-01	
Treg	FOXP3	0.027	3.94E-01		0.058	1.98E-01		0.393	4.15E-19	***
	CCR8	0.014	6.71E-01		0.157	4.61E-04	**	0.464	7.27E-27	***
	STAT5B	0.283	8.58E-20	***	0.505	4.67E-12	***	0.138	2.47E-03	*
	TGFb (TGFB1)	0.321	3.21E-25	***	0.198	9.43E-06	***	0.064	1.64E-01	
T cell exhaustion	PD-1 (PDCD1)	0.112	4.12E-04	**	0.051	2.56E-01		0.361	3.80E-16	***
	CTLA4	0.018	5.75E-01		0.081	7.27E-02	***	0.404	3.88E-20	***
	LAG3	−0.109	6.00E-04	**	−0.035	4.39E-01		0.212	3.11E-06	***
	TIM-3 (HAVCR2)	0.039	2.19E-01		0.213	1.78E-06	***	0.589	8.44E-46	***
	GZMB	0.056	7.82E-02		0.024	5.99E-01		0.267	3.33E-09	***

## Discussion

We systematically analyzed the expression levels of S1PR1 and the prognostic value in different types of cancers. Compared with levels in normal tissues, S1PR1 expression was significantly lower in BLCA, BRCA, CHOL, COAD, ESCA, HNSC, KICH, KIRP, LIHC, LUAD, LUSC, PRAD, READ, SKCM, STAD, and UCEC and was significantly higher in KIRC and THCA. Accordingly, S1PR1 expression patterns depend on the type of cancer. Prognostic data from Kaplan-Meier plotter showed that low levels of S1PR1 are significantly related to poor prognosis in breast cancer and lung cancer.

The down-regulation of *S1PR1* was associated with worse prognosis in breast cancer and lung cancer and was significantly related to clinical characteristics, such as gender, population, smoking status, and stage. These results suggested that S1PR1 is a prognostic biomarker in breast cancer and lung cancer. However, some literatures have reported the oncogenic role of S1PR1 in breast cancer. Lee H demonstrated that Stat3-induced S1PR1 expression, as well as S1P/S1PR1 pathway, is important for persistent Stat3 activation in cancer cells and the tumor microenvironment and for malignant progression [32]. This may be one of the molecular mechanisms by which S1PR1 mediates such a complex biological response. We considered that the main reason for this inconsistency is that our study analyzed the expression of S1PR1 at the overall level. We further verified the significant reduction of S1PR1 expression in breast cancer and lung cancer patients through TCGA analysis. Another study has also claimed a survival function benefit of S1P/S1PR signaling in BRCA patients, which might explain the obstacle to relative antagonist therapy in clinics [17]. A recent study determined that attenuated endothelial S1PR1 function led to increased tumor growth and metastasis, whereas S1PR1 overexpression led to smaller tumors, and strategies to enhance S1PR1 function in the tumor vasculature may potentiate the efficacy of cytotoxic and targeted anticancer therapies [33]. These studies support our findings that high expression of S1PR1 is beneficial for tumor survival.

The tumor microenvironment refers to non-cancer cells in and around tumors; infiltrated of immune cells in the tumor microenvironment plays a vital function in the occurrence and development of tumors [34, 35]. Lymphocyte infiltration in the tumor microenvironment is an independent predictor of cancer patient survival and lymph node metastasis [29, 30]. Studies have shown that S1PR1 can affect the proliferation and differentiation of lymphocytes in the tumor microenvironment [36]. The evaluation of immune cell infiltration in breast and lung cancers using the TIMER database revealed strong negative correlations between S1PR1 and tumor purity in BRCA, LUAD, and LUSC. Furthermore, the S1PR1 expression level was positively correlated with levels of CD8^+^ T, CD4^+^ T, neutrophils, macrophages, and DCs in BRCA. The correlation between S1PR1 expression and immune cell marker genes suggests that S1PR1 regulates lung cancer tumor immunity through multiple immune cell populations. These results indicate that high levels of S1PR1 could increase the cytotoxicity of the immune system and immune activation in BRCA, LUAD and LUSC by increasing the infiltration of CTLs, CD4 + T cells, and DCs. On the contrary, low expression of S1PR1 could lead to reduced infiltrated effector cells in the tumor microenvironment. As shown in recently reports, endothelial loss of S1PR1 led to a reduction in CD45+ cells, macrophages, and DCs, which influences tumor growth and metastasis [33]. In addition, S1P is involved in enhancing endocytosis and migration of mature dendritic cells through S1PR3, an event that may increase the immune response to cancer cells. Our findings are consistent with such reports, and these discoveries imply that S1PR1 plays an important role in recruiting and governing immune infiltration in BRCA, LUAD and LUSC.

To further elucidate the molecular mechanisms underlying the role of S1PR1 in breast and lung cancers, we used GSEA to identify pathways that are enriched in genes co-expressed with S1PR1. We found that S1PR1 was significantly associated with vasculogenesis, the purinergic receptor signaling pathway, and metabolism of nucleic acids in tumor conditions. This conclusion is consistent with previous research reports that showed that S1PR1 regulates vasculogenesis [7]. Recent studies have provided potential explanations for the associations between S1PR1 expression, immune infiltration, and poor prognosis. Angiogenesis mimicry (VM) system is a blood vessel-like network in which tumor cells are co-expressed with endothelial cells and tumor markers [37]. VM is closely related to a variety of human malignancies, including breast cancer [38]. Angiogenesis mimicry leads to worse prognosis, increased tumor metastasis, low 5-year overall survival, and increased mortality [39]. This shows that S1PR1 defects promote the occurrence of VM, and the knockout of S1PR1 in breast cancer cells increases the number of VMs. More importantly, tumor cells with low S1PR1 expression receive nutrition through VM, and accelerate tumor growth in animal models [40]. Recent research has shown that S1PR1 signaling is an important vascular factor affecting tumor progression, metastasis, and responses to chemotherapy and immunotherapy [33]. Strategies to enhance S1PR1 function in the tumor vasculature may enhance the cytotoxic killing effect and chemotherapy effect of targeted anti-cancer therapy.

A limitation of our study was the lack of in vitro and animal experiments to confirm the role of S1PR1 in the growth and progression of breast cancer and lung cancer and its relationship with the infiltration of immune cells in the tumor microenvironment. Therefore, further research is needed to verify the role of S1PR1 in breast cancer and lung cancer using these models.

## Conclusions

In conclusion, decreased S1PR1 expression was related to poor prognosis together with reduction of effect immune cell infiltration in breast and lung cancers. In addition, the down-regulation of S1PR1 may have profound effects on vasculogenic mimicry in tumor microenvironment. Accordingly, S1PR1 presumably plays a critical part in immune infiltration and acts as a prognostic marker in breast cancer and lung cancer.

## Supplementary information

**Additional file 1: Figure S1.** Prognostic potential of S1PR1 in different cancers. (a–c) Kaplan-Meier survival curves comparing the high and low expression of S1PR1 in soft tissue cancer (a), blood cancer (b), and brain cancer (c) in the PrognoScan. (d–f) Survival curves of gastric cancer analyzed with mRNA-seq data of TCGA of Kaplan-Meier plotter databases. OS = Overall survival; RFS = Relapse-Free Survival; PPS = Post-progression survival.

**Additional file 2: Table S1.** Correlations between S1PR1 and related genes and markers, as evaluated using GEPIA.

## Data Availability

The datasets used and/or analyzed during the current study are available from the corresponding author on reasonable request.

## Abbreviations

S1P: Sphingosine-1-phosphate
S1PR1: Sphingosine-1-phosphate receptor
CNV: Copy number variation
GO: Gene Ontology
BP: Biological processes
KEGG: Kyoto Encyclopedia of Genes and Genomes
GSEA: Gene set enrichment analysis
TCGA: The Cancer Genome Atlas
TIL: Tumor infiltrating lymphocytes
BLCA: Bladder urothelial carcinoma
BRCA: Breast invasive carcinoma
CHOL: Cholangiocarcinoma
COAD: Colon adenocarcinoma
ESCA: Esophageal carcinoma
HNSC: Head and neck squamous cell carcinoma
KICH: Kidney chromophobe
KIRP: Kidney renal papillary cell carcinoma
LIHC: Liver hepatocellular carcinoma
LUAD: Lung adenocarcinoma
LUSC: Lung squamous cell carcinoma
PRAD: Prostate adenocarcinoma
READ: Rectum adenocarcinoma
SKCM: Skin cutaneous melanoma
STAD: Stomach adenocarcinoma
UCEC: Uterine corpus endometrial carcinoma
KIRC: Kidney renal clear cell carcinoma
THCA: Thyroid carcinoma
OS: Overall survival
RFS: Relapse-Free Survival
PPS: Post-Progression survival
DSS: Disease-specific survival
DFS: Disease-free survival

## References

[1] Sun K, Zhang Y, D'Alessandro A, Nemkov T, Song A, Wu H (2016). Sphingosine-1-phosphate promotes erythrocyte glycolysis and oxygen release for adaptation to high-altitude hypoxia. Nat Commun.

[2] Asano Y, Stawski L, Hant F, Highland K, Silver R, Szalai G (2010). Endothelial Fli1 deficiency impairs vascular homeostasis: a role in scleroderma vasculopathy. Am J Pathol.

[3] Allende ML, Yamashita T, Proia RL (2003). G-protein-coupled receptor S1P1 acts within endothelial cells to regulate vascular maturation. Blood..

[4] Spiegel S, Milstien S (2011). The outs and the ins of sphingosine-1-phosphate in immunity. Nat Rev Immunol.

[5] Spiegel S, Milstien S (2002). Sphingosine 1-phosphate, a key cell signaling molecule. J Biol Chem.

[6] Proia RL, Hla T (2015). Emerging biology of sphingosine-1-phosphate: its role in pathogenesis and therapy. J Clin Invest.

[7] Sukocheva O, Wadham C, Gamble J, Xia P (2015). Sphingosine-1-phosphate receptor 1 transmits estrogens' effects in endothelial cells. Steroids..

[8] Sukocheva OA (2018). Expansion of Sphingosine kinase and Sphingosine-1-phosphate receptor function in Normal and Cancer cells: from membrane restructuring to mediation of estrogen signaling and stem cell programming. Int J Mol Sci.

[9] Rodriguez YI, Campos LE, Castro MG, Aladhami A, Oskeritzian CA, Alvarez SE (2016). Sphingosine-1 phosphate: a new modulator of immune plasticity in the tumor microenvironment. Front Oncol.

[10] Nakajima M, Nagahashi M, Rashid OM, Takabe K, Wakai T (2017). The role of sphingosine-1-phosphate in the tumor microenvironment and its clinical implications. Tumour Biol.

[11] Sukocheva OA, Furuya H, Ng ML, Friedemann M, Menschikowski M, Tarasov VV (2020). Sphingosine kinase and sphingosine-1-phosphate receptor signaling pathway in inflammatory gastrointestinal disease and cancers: a novel therapeutic target. Pharmacol Ther.

[12] Folkman J (2007). Angiogenesis: an organizing principle for drug discovery?. Nat Rev Drug Discov.

[13] Ferrara N (2010). Pathways mediating VEGF-independent tumor angiogenesis. Cytokine Growth Factor Rev.

[14] Jung B, Obinata H, Galvani S, Mendelson K, Ding B, Skoura A (2012). Flow-regulated endothelial S1P receptor-1 signaling sustains vascular development. Dev Cell.

[15] Gaengel K, Niaudet C, Hagikura K, Hagikura K, Laviña B, Muhl L (2012). The sphingosine-1-phosphate receptor S1PR1 restricts sprouting angiogenesis by regulating the interplay between VE-cadherin and VEGFR2. Dev Cell.

[16] Xiao S, Yang J (2019). Preclinical study of the antitumor effect of sphingosine-1-phosphate receptor 1 antibody (S1PR_1_-antibody) against human breast cancer cells. Investig New Drugs.

[17] Lei FJ, Cheng BH, Liao PY (2018). Survival benefit of sphingosin-1-phosphate and receptors expressions in breast cancer patients. Cancer Med.

[18] Glinsky GV, Glinskii AB, Stephenson AJ, Hoffman RM, Gerald WL (2004). Gene expression profiling predicts clinical outcome of prostate cancer. J Clin Invest.

[19] Chen B, Tang H, Chen X, Zhang G, Wang Y, Xie X (2018). Transcriptomic analyses identify key differentially expressed genes and clinical outcomes between triple-negative and non-triple-negative breast cancer. Cancer Manag Res.

[20] Tang H, Huang X, Wang J, Yang L, Kong Y, Gao G (2019). circKIF4A acts as a prognostic factor and mediator to regulate the progression of triple-negative breast cancer. Mol Cancer.

[21] Rhodes DR, Kalyana-Sundaram S, Mahavisno V, Varambally R, Yu J, Briggs BB (2007). Oncomine 3.0: genes, pathways, and networks in a collection of 18,000 cancer gene expression profiles. Neoplasia..

[22] Lánczky A, Nagy Á, Bottai G, Munkácsy G, Szabó A, Santarpia L (2016). miRpower: a web-tool to validate survival-associated miRNAs utilizing expression data from 2178 breast cancer patients. Breast Cancer Res Treat.

[23] Gao J, Aksoy BA, Dogrusoz U, Dresdner G, Gross B, Sumer SO (2013). Integrative analysis of complex cancer genomics and clinical profiles using the cBioPortal. Sci Signal.

[24] Szász AM, Lánczky A, Nagy Á, Förster S, Hark K, Green JE (2016). Cross-validation of survival associated biomarkers in gastric cancer using transcriptomic data of 1,065 patients. Oncotarget..

[25] Li T, Fan J, Wang B, Traugh N, Chen Q, Liu JS (2017). TIMER: a web server for comprehensive analysis of tumor-infiltrating immune cells. Cancer Res.

[26] Tang Z, Li C, Kang B, Gao G, Li C, Zhang Z (2017). GEPIA: a web server for cancer and normal gene expression profiling and interactive analyses. Nucleic Acids Res.

[27] Vasaikar SV, Straub P, Wang J, Zhang B (2018). LinkedOmics: analyzing multi-omics data within and across 32 cancer types. Nucleic Acids Res.

[28] Chandrashekar DS, Bashel B, Balasubramanya SAH, Creighton CJ, Ponce-Rodriguez I, Chakravarthi BVSK (2017). UALCAN: a portal for facilitating tumor subgroup gene expression and survival analyses. Neoplasia..

[29] Ohtani H (2007). Focus on TILs: prognostic significance of tumor infiltrating lymphocytes in human colorectal cancer. Cancer Immun.

[30] Azimi F, Scolyer RA, Rumcheva P, Moncrieff M, Murali R, McCarthy SW (2012). Tumor-infiltrating lymphocyte grade is an independent predictor of sentinel lymph node status and survival in patients with cutaneous melanoma. J Clin Oncol.

[31] Yoshihara K, Shahmoradgoli M, Martínez E, Vegesna R, Kim H, Torres-Garcia W (2013). Inferring tumour purity and stromal and immune cell admixture from expression data. Nat Commun.

[32] Lee H, Deng J, Kujawski M, Yang C, Liu Y, Herrmann A (2010). STAT3-induced S1PR1 expression is crucial for persistent STAT3 activation in tumors. Nat Med.

[33] Cartier A, Leigh T, Liu CH, Hla T (2020). Endothelial sphingosine 1-phosphate receptors promote vascular normalization and antitumor therapy. Proc Natl Acad Sci U S A.

[34] Zhu J, Petit PF, Van den Eynde BJ (2019). Apoptosis of tumor-infiltrating T lymphocytes: a new immune checkpoint mechanism. Cancer Immunol Immunother.

[35] Sharma P, Hu-Lieskovan S, Wargo JA, Ribas A (2017). Primary, adaptive, and acquired resistance to Cancer immunotherapy. Cell..

[36] Aoki M, Aoki H, Ramanathan R, Hait NC, Takabe K (2016). Corrigendum to "Sphingosine-1-phosphate signaling in immune cells and inflammation: roles and therapeutic potential". Mediat Inflamm.

[37] Valdivia A, Mingo G, Aldana V, Pinto MP, Ramirez M, Retamal C (2019). Fact or fiction, it is time for a verdict on Vasculogenic mimicry?. Front Oncol.

[38] El Hallani S, Boisselier B, Peglion F, Rousseau A, Colin C, Idbaih A (2010). A new alternative mechanism in glioblastoma vascularization: tubular vasculogenic mimicry. Brain..

[39] Cao Z, Bao M, Miele L, Sarkar FH, Wang Z, Zhou Q (2013). Tumour vasculogenic mimicry is associated with poor prognosis of human cancer patients: a systemic review and meta-analysis. Eur J Cancer.

[40] Liu S, Ni C, Zhang D, Sun H, Dong X, Che N (2019). S1PR1 regulates the switch of two angiogenic modes by VE-cadherin phosphorylation in breast cancer. Cell Death Dis.

