# Supplementary material for: Prognostic value of S1PR1 and its correlation with immune infiltrates in breast and lung cancers
Source: BMC Cancer. 2020 Aug 15;20:766. doi: 10.1186/s12885-020-07278-2 (PMC7429796; doi:10.1186/s12885-020-07278-2)
Supplement: Supplementary file 1 — Additional file 1: Figure S1. Prognostic potential of S1PR1 in different cancers. (a–c) Kaplan-Meier survival curves comparing the high and low expression of S1PR1 in soft tissue cancer (a), blood cancer (b), and brain cancer (c) in the PrognoScan. (d–f) Survival curves of gastric cancer analyzed with mRNA-seq data of TCGA of Kaplan-Meier plotter databases. OS = Overall survival; RFS = Relapse-Free Survival; PPS = Post-progression survival. [file 12885_2020_7278_MOESM1_ESM.pptx]

## Slide 1
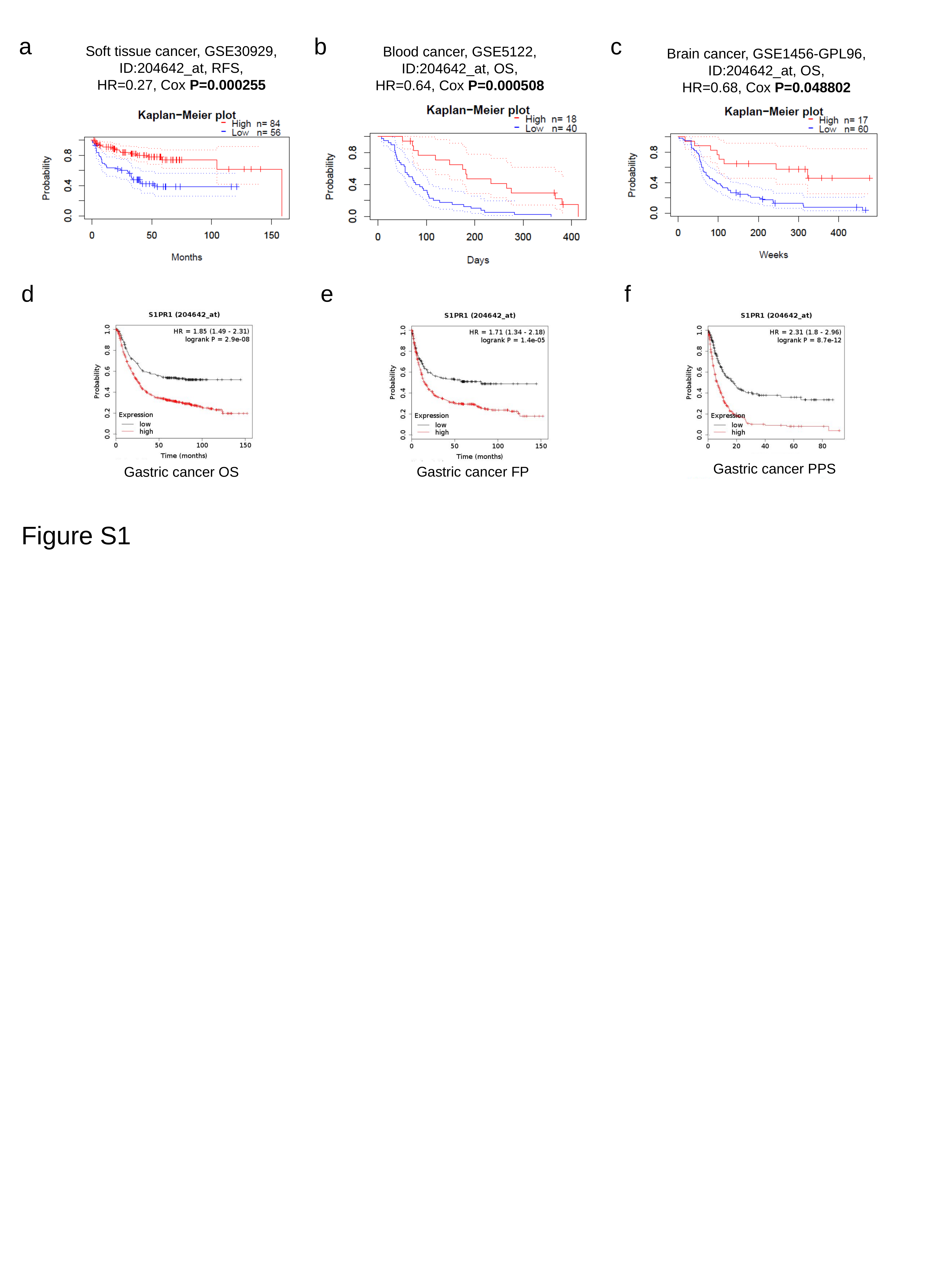

a
b
c
Soft tissue cancer, GSE30929, ID:204642_at, RFS,
HR=0.27, Cox P=0.000255
Blood cancer, GSE5122, ID:204642_at, OS,
HR=0.64, Cox P=0.000508
Brain cancer, GSE1456-GPL96, ID:204642_at, OS,
HR=0.68, Cox P=0.048802
d
e
f
Gastric cancer OS
Gastric cancer FP
Gastric cancer PPS
Figure S1
