# Supplementary material for: Prognostic value of S1PR1 and its correlation with immune infiltrates in breast and lung cancers
Source: BMC Cancer. 2020 Aug 15;20:766. doi: 10.1186/s12885-020-07278-2 (PMC7429796; doi:10.1186/s12885-020-07278-2)
Supplement: Supplementary file 2 — Additional file 2: Table S1. Correlations between S1PR1 and related genes and markers, as evaluated using GEPIA. [file 12885_2020_7278_MOESM2_ESM.docx]

**Sup table 1.** Correlations between S1PR1 and related genes and markers, as evaluated using GEPIA

|  |  | BRAC | | | LUAD | | | LUSC | | |
| --- | --- | --- | --- | --- | --- | --- | --- | --- | --- | --- |
| Description | Gene markers | Purity | |  | Purity | |  | Purity | |  |
|  | varX | cor | p | | cor | p | | cor | p | |
| CD8+ T cell | CD8A | 0.46 | 1.10E-57 | *** | 0.26 | 4.10E-09 | *** | 0.23 | 4.10E-07 | *** |
|  | CD8B | 0.37 | 1.20E-35 | *** | 0.19 | 3.00E-05 | *** | 0.052 | 2.50E-01 |  |
| T cell (general) | CD3D | 0.39 | 2.10E-40 | *** | 0.21 | 2.20E-06 | *** | 0.35 | 8.90E-16 | *** |
|  | CD3E | 0.46 | 6.80E-28 | *** | 0.36 | 2.50E-16 | *** | 0.43 | 0.00E+00 | *** |
|  | CD2 | 0.4 | 5.30E-44 | *** | 0.3 | 9.90E-12 | *** | 0.37 | 0.00E+00 | *** |
| B cell | CD19 | 0.35 | 3.10E-32 | *** | 0.27 | 1.80E-09 | *** | 0.33 | 3.60E-14 | *** |
|  | CD79A | 0.36 | 2.60E-34 | *** | 0.25 | 1.90E-08 | *** | 0.36 | 0.00E+00 | *** |
| Monocyte | CD86 | 0.3 | 1.20E-23 | *** | 0.41 | 2.30E-21 | *** | 0.53 | 0.00E+00 | *** |
|  | CD115 (CSF1R) | 0.45 | 2.80E-55 | *** | 0.44 | 2.60E-24 | *** | 0.47 | 0.00E+00 | *** |
| TAM | CCL2 | 0.28 | 1.10E-21 | ** | 0.27 | 1.30E-09 | *** | 0.33 | 9.00E+00 | *** |
|  | CD68 | 0.27 | 3.30E-19 | *** | 0.45 | 6.60E-25 | *** | 0.39 | 0.00E+00 | *** |
|  | IL10 | 0.32 | 1.90E-26 | *** | 0.39 | 8.30E-19 | *** | 0.34 | 9.30E-15 | *** |
| M1 Macrophage | INOS (NOS2) | 0.32 | 1.60E-27 | *** | 0.48 | 3.30E-29 | *** | -0.064 | 1.60E-01 |  |
|  | IRF5 | 0.18 | 1.00E-09 |  | 0.18 | 5.30E-05 | *** | -0.0067 | 8.80E-01 |  |
|  | COX2(PTGS2) | 0.46 | 1.70E-57 | *** | 0.14 | 2.30E-03 | * | 0.097 | 3.30E-02 |  |
| M2 Macrophage | CD163 | 0.23 | 1.20E-14 | *** | 0.44 | 2.00E-24 | *** | 0.54 | 0.00E+00 | *** |
|  | VSIG4 | 0.29 | 4.10E-22 | *** | 0.39 | 3.30E-19 | *** | 0.47 | 0.00E+00 | *** |
|  | MS4A4A | 0.43 | 7.40E-50 | *** | 0.49 | 4.50E-31 | *** | 0.5 | 0.00E+00 | *** |
| Neutrophils | CD66b (CEACAM8) | 0.032 | 2.90E-01 |  | 0.3 | 1.50E-11 | *** | 0.3 | 2.30E-11 | *** |
|  | CD11b (ITGAM) | 0.27 | 3.40E-20 | *** | 0.4 | 1.50E-19 | *** | 0.27 | 1.20E-09 | *** |
|  | CCR7 | 0.49 | 1.50E-66 | *** | 0.47 | 4.40E-28 | *** | 0.36 | 2.20E-16 | *** |
| Natural killer cell | KIR2DL1 | 0.19 | 3.50E-10 | *** | 0.27 | 9.50E-10 | *** | 0.14 | 1.40E-03 | * |
|  | KIR2DL3 | 0.18 | 1.70E-09 | *** | 0.24 | 8.20E-08 | ** | 0.12 | 8.60E-03 | * |
|  | KIR2DL4 | 0.11 | 5.10E-04 | ** | 0.41 | 3.80E-02 |  | 0.03 | 5.10E-01 |  |
|  | KIR3DL1 | 0.23 | 1.40E-14 | *** | 0.24 | 9.20E-05 | *** | 0.26 | 4.10E-09 | *** |
|  | KIR3DL2 | 0.24 | 3.00E-16 | *** | 0.21 | 2.70E-06 | *** | 0.13 | 4.00E-03 | * |
|  | KIR3DL3 | 0.05 | 1.00E-01 |  | -0.015 | 7.30E-01 |  | 0.029 | 5.20E-01 |  |
|  | KIR2DS4 | 0.16 | 2.10E-07 | *** | 0.2 | 1.20E-05 | *** | 0.16 | 4.40E-04 | ** |
| Dendritic cell | HLA-DPB1 | 0.46 | 4.80E-57 | *** | 0.39 | 6.10E-19 | *** | 0.56 | 0.00E+00 | *** |
|  | HLA-DQB1 | 0.18 | 1.10E-09 | *** | 0.14 | 2.60E-03 |  | 0.25 | 2.80E-08 | *** |
|  | HLA-DRA | 0.39 | 1.10E-40 | *** | 0.26 | 5.30E-09 | *** | 0.47 | 0.00E+00 | ** |
|  | HLA-DPA1 | 0.43 | 4.70E-50 | *** | 0.28 | 4.30E-10 | *** | 0.49 | 0.00E+00 | ** |
|  | BDCA-1(CD1C) | 0.6 | 6.60E-106 | *** | 0.19 | 2.90E-05 | *** | 0.48 | 0.00E+00 | *** |
|  | BDCA-4(NRP1) | 0.6 | 4.70E-106 | *** | 0.17 | 1.50E-04 | ** | 0.32 | 2.40E-13 | *** |
|  | CD11c (ITGAX) | 0.34 | 5.10E-30 | *** | 0.3 | 1.40E-11 | *** | 0.49 | 0.00E+00 | *** |
| Th1 | T-bet (TBX21) | 0.43 | 6.60E-49 | *** | 0.021 | 6.50E-01 | *** | 0.4 | 0.00E+00 | *** |
|  | STAT4 | 0.49 | 6.60E-68 | *** | 0.15 | 9.00E-04 | *** | 0.45 | 0.00E+00 | *** |
|  | STAT1 | 0.1 | 5.50E-04 | ** | 0.066 | 1.50E-01 |  | 0.08 | 7.90E-02 |  |
|  | IFN-g (IFNG) | 0.17 | 7.60E-09 | *** | -0.036 | 4.30E-01 |  | 0.01 | 8.30E-01 |  |
|  | TNF-a (TNF) | -0.035 | 2.40E-01 |  | 0.097 | 3.30E-02 |  | 0.051 | 2.60E-01 |  |
| Th2 | GATA3 | 0.042 | 1.70E-01 |  | -0.023 | 6.10E-01 |  | 0.054 | 2.30E-01 |  |
|  | STAT6 | 3.70E-37 | 6.69E-13 | *** | 0.13 | 4.70E-03 | * | 0.092 | 4.30E-02 |  |
|  | STAT5A | 0.38 | 2.20E-39 | *** | 0.44 | 0.00E+00 | *** | 0.37 | 0.00E+00 | *** |
|  | IL13 | 0.15 | 4.80E-07 | *** | 0.076 | 9.40E-02 |  | 0.28 | 7.00E-10 | *** |
| Tfh | BCL6 | 0.31 | 1.10E-25 | *** | 0.17 | 1.90E-04 | ** | 0.087 | 5.50E-02 |  |
|  | IL21 | 0.18 | 6.20E-09 | *** | 0.11 | 1.70E-02 |  | 0.21 | 3.20E-06 | *** |
| Th17 | STAT3 | 0.26 | 1.00E-18 | *** | 0.24 | 6.20E-08 | *** | 0.17 | 1.40E-04 | ** |
|  | IL17A | 0.04 | 1.80E-01 |  | 0.041 | 3.70E-01 |  | -0.042 | 3.60E-01 |  |
| Treg | FOXP3 | 0.2 | 2.50E-11 | *** | 0.2 | 7.70E-06 | *** | 0.31 | 1.40E-12 | *** |
|  | CCR8 | 0.22 | 3.90E-13 | *** | 0.26 | 3.90E-09 | *** | 0.29 | 1.30E-10 | *** |
|  | STAT5B | 0.42 | 2.00E-46 | *** | 0.4 | 0.00E+00 | *** | 0.29 | 4.00E-11 | *** |
|  | TGFb (TGFB1) | 0.47 | 1.10E-59 | *** | 0.23 | 2.30E-07 | *** | 0.06 | 1.90E-01 |  |
| T cell exhaustion | PD-1 (PDCD1) | 0.3 | 1.20E-24 | ** | 0.11 | 1.30E-02 |  | 0.35 | 8.90E-16 | *** |
|  | CTLA4 | 0.22 | 1.40E-13 | *** | 0.2 | 1.20E-05 | *** | 0.34 | 8.90E-15 | *** |
|  | LAG3 | -0.0072 | 8.10E-01 |  | 0.0036 | 9.40E-01 |  | 0.094 | 3.90E-02 |  |
|  | TIM-3 (HAVCR2) | 0.28 | 6.10E-21 | *** | 0.34 | 8.40E-15 | *** | 0.46 | 0.00E+00 | *** |
|  | GZMB | 0.21 | 3.60E-12 | *** | 0.022 | 6.30E-01 |  | 0.14 | 2.60E-03 | * |
